# Exploring the Prevalence of *SMN1* Duplication and Deletion in Russia and Its Impact on Carrier Screening

**DOI:** 10.3390/ijms26051984

**Published:** 2025-02-25

**Authors:** Kristina Mikhalchuk, Aleksander Polyakov, Viktoria V. Zabnenkova, Olga Ismagilova, Olga Shchagina

**Affiliations:** Research Centre for Medical Genetics, 1 Moskvorechye St., Moscow 115522, Russiaschagina@med-gen.ru (O.S.)

**Keywords:** spinal muscular atrophy, silent carrier, genotype (2 + 0), carrier screening, 5q SMA, *SMN1*, duplication, deletion, markers, c.*3+80T>G, c.*211_*212del

## Abstract

5q spinal muscular atrophy (5q SMA) is one of the most prevalent autosomal recessive disorders worldwide. In 5q SMA, cases of silent carriers have been reported, including *SMN1* duplications, intragenic subtle variants, de novo variants, and mosaicism. This study included DNA samples from 3412 unexamined unrelated individuals with no known family history of 5q SMA. In addition, we studied 15 families in which the children had a confirmed diagnosis of 5q SMA caused by a homozygous deletion of exon 7 of *SMN1*. Each family included one parent who was a carrier of a heterozygous deletion of *SMN1*, while the other parent had two copies of *SMN1*. The copy number of *SMN1* and *SMN2* was detected by MLPA. Two previously reported genetic markers of *SMN1* duplication, c.*3+80T>G and c.*211_*212del, were tested in 143 Russian residents with three copies of *SMN1* and 15 parents with two copies of *SMN1*. The frequency of a heterozygous carrier of exon 7 deletion of *SMN1* is 1 in 36 individuals (95% CI 33 to 39). The frequency of exon 7 duplication of *SMN1* is 1 in 25 individuals (95% CI 20 to 30). Only three individuals of the studied *SMN1* duplication carriers were detected to have genetic markers of *SMN1* duplication. The study of *SMN1* duplication genetic markers (c.*3+80T>G and c.*211_*212del) in Russian residents reveals only 1.9% of *SMN1* duplication carriers.

## 1. Introduction

5q spinal muscular atrophy (5q SMA) is considered one of the most common autosomal recessive diseases worldwide. The prevalent pathogenic variant associated with 5q SMA is a deletion involving exon 7 of *SMN1*, which is present in 99% of the chromosomes of affected individuals and in a homozygous state accounts for 95–98% of 5q SMA cases [1]. The ability to determine the genetic cause of 5q SMA is critically important for patients, as only those with biallelic variants in *SMN1* are eligible for genetic therapy. The accurate identification of pathogenic variants in patients with 5q SMA is also essential for genetic counseling in affected families. Currently, three FDA-approved treatment modalities for 5q SMA are available: (1) the gene therapy Onasemnogene abeparvovec (marketed as Zolgensma^®^ (NOVARTIS PHARMA, AG (Basel, Switzerland)), which delivers a functional *SMN1* to cells via an adeno-associated viral vector; (2) the antisense oligonucleotide (ASO) Nusinersen (marketed as Spinraza® BIOGEN NETHERLANDS, B.V. (Badhoevedorp, The Netherlands)), which increases levels of mRNA containing exon 7, produced by *SMN2*, by displacing splicing repressors hnRNPA1/A2 from their binding site; and (3) the small molecule splicing modifier *SMN2* Risdiplam (marketed as Evrysdi^®^ F. Hoffmann-La Roche, Ltd. (Basel, Switzerland)) [2,3].

Screening for exon 7 deletion of *SMN1* as the underlying genetic cause of 5q SMA should be recommended to all couples during preconception counseling and as part of screening programs for potential carriers of 5q SMA. The estimated carrier frequency of 5q SMA ranges from 1 in 25 to 1 in 50 in different ethnic groups [4]. Due to the high carrier prevalence and severity of the disease, the American College of Medical Genetics and Genomics (ACMG) recommends pan ethnic screening for 5q SMA among all couples [5].

**Figure 1:**
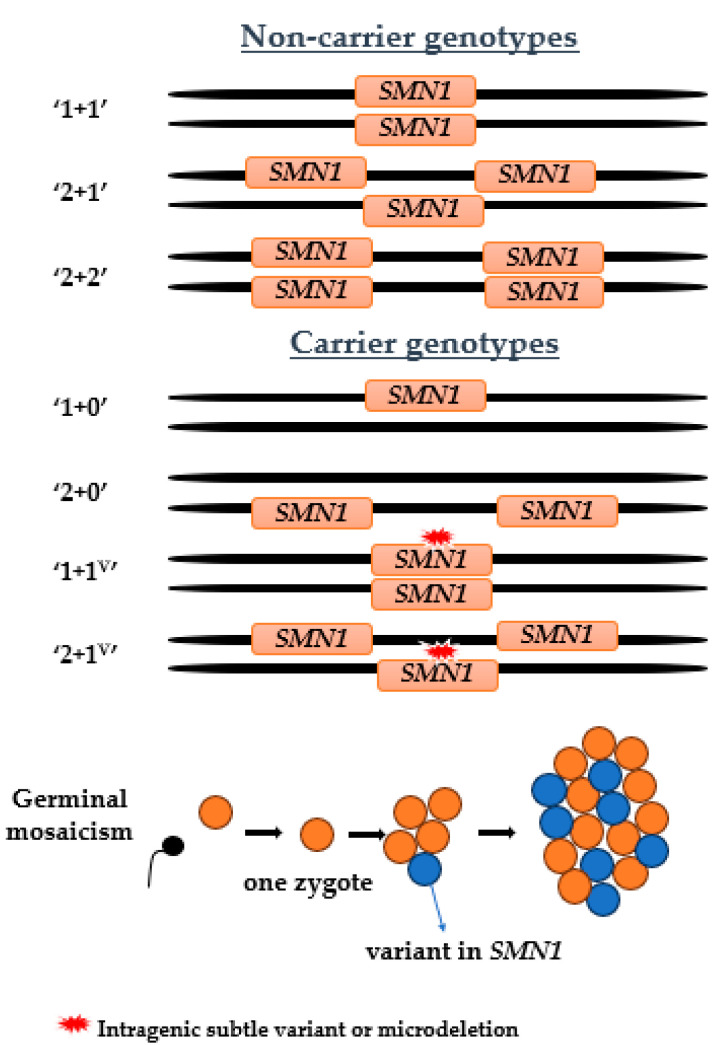
Genotype variants in individuals without 5q SMA. Non-carriers carry at least one copy of *SMN1* on each chromosome (genotypes 1 + 1, 2 + 1, 2 + 2). The majority of carriers have only one copy of *SMN1*, with no *SMN1* on the other chromosome (genotype 1 + 0). Silent carriers have two copies of *SMN1* on one chromosome, while *SMN1* is absent on the other chromosome (genotype 2 + 0), which is not identified by routine quantitative methods. Silent carriers may also have two copies of *SMN1* on each of the chromosomes, but one of the copies may carry an intragenic subtle variant (genotypes 1 + 1^V^, 2 + 1^V^). In addition, quantitative carrier screening is unable to detect germline mosaicism by variants in *SMN1*.

Most carriers of 5q SMA have a genotype characterized by one deletion of *SMN1* on one chromosome and one functional copy of *SMN1* on the other (genotype 1 + 0) (Figure 1). Identification of *SMN1* copy number in population-based carrier screening allows the detection of most couples who are heterozygous carriers of *SMN1* deletion before the birth of a child with 5q SMA. The efficiency of detection of heterozygous carriers by quantitative *SMN1* copy number testing ranges from 70.5% to 95% in various ethnic groups [4,6]. Difficulties in accurately determining carrier status result from a number of complexities related to 5q SMA, including the presence of intragenic subtle variants in *SMN1*, the genotype in which two copies of *SMN1* are present on the one chromosome with a deletion on the homologous chromosome, and the possibility of germline mosaicism, which may hinder the detection of carrier status in available genetic materials.

To date, two genetic markers associated with *SMN1* duplication have been reported in the literature: variants NM_000344.3:c.*3+80T>G (g.27134T>G) (rs143838139), located in intron 7, and c.*211_*212del (g.27706_27707delAT), located in exon 8 of *SMN1* [4]. Original studies of genetic markers of *SMN1* duplication focused on the Ashkenazi Jewish population, where a founder effect from a common ancestor was hypothesized for the allele associated with *SMN1* duplication. In the Ashkenazi Jewish population, the carrier frequency of 5q SMA is estimated to be approximately 1 in 41 individuals. Detection by copy number analysis of *SMN1* is estimated to be approximately 90%. Of the remaining 10%, about 8% are identified as silent carriers of an *SMN1* deletion on one allele (genotype 2 + 0). Several founder haplotypes of *SMN1* have been identified in Ashkenazi Jewish individuals, in particular one that accounts for approximately 50% of all *SMN1* duplications (genotype 2 + 1) in this ethnic group. The sequencing of the *SMN1* genomic region among these carriers revealed significant variants, including c.3*+80T>G and c.*211_*212del, as well as other single nucleotide polymorphisms (SNPs) characteristic of this haplotype. In comparison, the prevalence of the *SMN1* duplication (genotype 2 + 1) is significantly higher in the African American population at 49% [4]. This increased frequency correlates with the large number of asymptomatic carriers of the *SMN1* deletion (genotype 2 + 0), and they were found to have the same unique haplotype as Ashkenazi Jewish carriers.

The prevalence of *SMN1* duplications in Russia has not yet been studied. In this study, we analyzed the frequency of *SMN1* duplications in unexamined unrelated individuals in Russia with no known family history of 5q SMA. Additionally, we investigated the diagnostic value of using the *SMN1* duplication genetic markers (c.*3+80T>G and c.*211_*212del) to identify silent *SMN1* deletion carriers. Understanding these factors is crucial for improving genetic screening strategies for 5q SMA in the Russian population.

## 2. Results

This study presents a quantitative copy number analysis of exon 7 copy number of *SMN1* among 3412 unexamined unrelated residents of Russia with no known family history of 5q SMA. The analysis included a total of 6824 chromosomes, and further details are presented in Table 1.

In a cohort of 94 residents of Russia, a heterozygous deletion of exon 7 of *SMN1* was identified, which resulted in an allelic frequency of 0.014 (Appendix B). This represents a carrier frequency of approximately 1 in 36 residents (95% CI 33 to 39).

In a study of Russian population genotypes, more than two copies of exon 7 of *SMN1* were detected in 143 residents. Notably, three copies were detected in 137 participants. Obviously, this genotype indicates duplication of *SMN1* on one homologous chromosome 5, which resulted in a frequency of 4% in the study cohort. Based on the principles of Hardy–Weinberg equilibrium, we expected to see five to six individuals homozygous for this duplication (Appendix B). In fact, six individuals carried four copies of exon 7 of *SMN1*, confirming their homozygous status for *SMN1* duplication. Thus, the duplication of exon 7 of *SMN1* was detected in 149 out of 6824 chromosomes, which resulted in an allele frequency of 0.022.

Based on the allelic frequency of *SMN1* deletion and duplication among Russian residents, the frequency of silent carriers, characterized by the genotype 2 + 0 (in which one chromosome 5 carries deletion of *SMN1* while the other chromosome carries duplication of *SMN1*), is 0.0006, or 1 in 1667 residents of Russia (Appendix B). Thus, the findings reveal that 99.9% of Russian residents who requested to be screened for 5q SMA with two copies of exon 7 of *SMN1* determined by MLPA are in fact not silent carriers of *SMN1* deletion as a result of the genotype 2 + 0.

This study analyzed the presence of genetic markers of *SMN1* duplication, c.*3+80T>G and c.*211_*212del, in a cohort of 158 Russian residents carrying *SMN1* du-plication (population cohort and 15 parents of 5q SMA patients with two copies of *SMN1*), as well as in 209 unrelated Russian residents with one copy of *SMN1*. A control group not carrying *SMN1* duplication was also examined for these markers. Among the 158 individuals with *SMN1* duplication, two were identified to carry the genetic markers c.*3+80T>G and c.*211_*212del. Furthermore, one additional carrier of *SMN1* duplication and these genetic markers was identified among the 15 investigated parents of 5q SMA patients. Notably, the genetic markers c.*3+80T>G and c.*211_*212del were absent in all samples within the control cohort consisting 209 individuals with one copy of *SMN1*. Thus, the identification of the genetic markers c.*3+80T>G and c.*211_*212del indicates that only 1.9% of carriers of *SMN1* duplication are identifiable using these markers in the Russian population. Therefore, the detection of these genetic markers for the identification of silent carriers of 5q SMA due to genotype 2 + 0 is uninformative in Russia.

**Figure 2:**
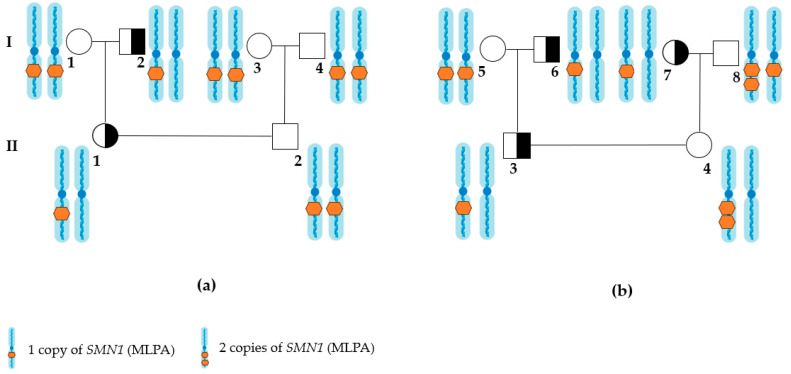
Example of allele distribution with *SMN1* in two families (**a**,**b**). In the first pedigree example (**a**), individuals requested carrier screening for a deletion in exon 7 of *SMN1*, individuals with two copies of *SMN1* by MLPA (I.1, I.3, I.4, II.2) carry two copies of *SMN1* on two alleles. In the second pedigree example (**b**), some individuals with two copies of *SMN1* by MLPA (I.5, II.4) can carry two copies of *SMN1* on the one allele (silent carriers of *SMN1* deletion). Thus, when one partner is identified as a carrier of *SMN1* deletion (II.3) and the other partner has two copies of *SMN1* (II.4), it is recommended to determine the copy number of *SMN1* in the parents of the partner with two copies of the *SMN1*. Individuals (I.2, I.6, I.7, I.8, II.1) carry one copy of *SMN1* by MLPA. Chromosome 5 is schematically represented in blue, while the number of *SMN1* copies, detected by MPLA, is schematically represented in orange. In this representation, one orange hexagon schematically represents one copy of *SMN1*, and two hexagons represent two copies of *SMN1*.

The issue of silent carrying is apparent for the partners of deletion carriers. When determining the necessity for further genetic tests, it is crucial to acknowledge the exceedingly low probability—approximately 0.1%—that an individual with two copies of *SMN1* could have the genotype 2 + 0. In scenarios involving couples where one partner is a known carrier of a pathogenic variant in *SMN1*, while the other carries two copies of *SMN1*, it is possible to test for the genetic markers of *SMN1* duplication as well as copy number analyses of *SMN1* in the relatives of the partner carrying two copies of *SMN1*. Such a family analysis may provide insights regarding the cis or trans configuration of the existing two copies of *SMN1*, when *SMN1* duplication markers are absent. For instance, the presence of three *SMN1* copies in one parent of a consulted individual with two copies of *SMN1*, contrasted with one copy of *SMN1* in the other parent, could indicate the possibility of silent carrier status in the offspring (Figure 2). Unfortunately, it is important to note that the absence of deletions and duplications in *SMN1* among the parents does not eliminate the possibility of an individual carrying a genotype 2 + 0.

## 3. Discussion

Homo sapiens is the only species with two *SMN* paralogs in its genome: *SMN1* and *SMN2*; all other species have only one *SMN*. In primates, only one *SMN* copy has been identified, indicating that the duplication of *SMN* and the surrounding genes of the SMN locus on chromosome 5q13.2 likely occurred during the evolutionary transition from primates to humans. Notably, while undergoing evolutionary changes, the nucleotide sequence of the duplicated *SMN* copy underwent important alterations, resulting in the formation of the *SMN2* paralog. Studies show significant differences in the SMN locus among various ethnic groups. In particular, among African Americans, the frequency of carrying the genotype when both copies of *SMN1* on one chromosome in a cis configuration is eight times greater than the frequency of *SMN1* duplication observed in Caucasians. In contrast, Caucasians predominantly present a genotype with one copy of *SMN1* and one copy of *SMN2* [7].

Thus, in certain individuals who carry the deletion of exon 7 of *SMN1*, two copies of *SMN1* may be localized on one chromosome in a cis configuration, resulting in a genotype 2 + 0. This configuration reduces the sensitivity of many carrier testing methodologies that measure only the number of copies. The mechanism of the origin of *SMN1* duplication has not yet been thoroughly investigated; however, it may involve mechanisms similar to those that lead to the duplication of *SMN2* on one chromosome: (1) gene conversion; (2) unequal crossing-over; and (3) a two-step process, which includes the intrachromosomal deletion of *SMN2* followed by the duplication of the remaining *SMN1* [8].

This study identifies the frequency of heterozygous carriers of the exon 7 deletion of *SMN1* among Russian residents which is 1 in 36 individuals (95% CI 33 to 39). In comparison, the carrier frequency among other ethnic groups ranges from 1 in 25 to 1 in 50 [4]. Furthermore, the frequency of *SMN1* duplication varies depending on ethnicity and exhibits variability across different populations (Table 2).

The frequency of carriers of *SMN1* duplication among residents of Russia is lower than that observed in various ethnic cohorts in the United States (χ^2^ = 25.307, *p* < 0.001) and in Ashkenazi Jews in Israel (χ^2^ = 4.825, *p* = 0.029), but is higher than that in residents of Singapore (χ^2^ = 4.253, *p* = 0.04).

The literature documents a limited number of studies on the occurrence of the genetic markers of *SMN1* duplication among various populations, as well as studies of families in which one parent of a patient with 5q SMA carries two copies of *SMN1* in their genome (Table 3).

The genetic markers c.*3+80T>G and c.*211_*212del present on chromosomes with two copies of *SMN1* in cis configuration are detected with varying frequencies among different ethnic groups: 11 of 61 residents in Spain carrying 3–4 copies of *SMN1*, 113 of 135 African Americans, 3 of 24 Asians, 22 of 42 Hispanics, and 6 of 31 Caucasians (x^2^ = 37.2, *p* < 0.001). In contrast, these genetic markers were detected in only two of 143 residents of Russia carrying 3–4 copies of *SMN1*. Furthermore, in studies involving parent groups carrying two copies of *SMN1* who have children with a homozygous deletion of *SMN1*, the genetic markers c.*3+80T>G and c.*211_*212del were also detected significantly less frequently among residents of Russia compared to those from Spain and Singapore [4,9,10].

In individuals carrying one copy of *SMN1* from various ethnic cohorts, a haplotype of these two SNPs was detected in one African American [4]. The presence of these genetic markers on a chromosome without *SMN1* duplication reduces their value as diagnostic markers. Specifically, the use the genetic markers c.*3+80T>G and c.*211_*212del as a marker of *SMN1* duplication could result in both false-negative results— in cases of *SMN1* duplication and absence of the genetic markers —and false-positive results.

The genetic markers c.*3+80T>G and c.*211_*212del are more commonly observed within the haplotype. However, alleles with *SMN1* duplication that carry only the c.*3+80T>G variant have been identified in individuals from Singapore and Spain [9,10]. Notably, the c.*3+80T>G variant is preferred as a molecular marker for detecting *SMN1* duplication than c.*211_*212del. The low frequency of marker SNPs on chromosomes with *SMN1* duplication among cohorts of Asian and Caucasian cohorts in the United States, as well as residents of Singapore and Russia, can be attributed to the fact that new mutational changes at the SMN locus may also contribute to the occurrence of *SMN1* duplication.

This study had a notable limitation concerning the geographical representation of residents of Russia. The majority of participants were concentrated in Moscow, the Moscow region, and surrounding areas, indicating that these regions are overrepresented, which may affect the generalizability of the findings. It is important to note that the central regions of Russia have a higher population density than more distant regions.

## 4. Materials and Methods

### 4.1. The Cohort of the Present Study

This study included DNA samples from 3412 unexamined unrelated individuals (bank of the DNA diagnostic laboratory of the Research Center for Medical Genetics) with no known family history of 5q SMA residing in 39 regions of Russia. Individuals requested carrier screening for recessive disorders for preconception prevention for birth planning at the Research Center for Medical Genetics between 2011 and 2023. A limitation of this study was the lack of equal sampling of residents of all regions, so the majority of participants were from Moscow, the Moscow Region, and neighboring regions.

This study also included DNA samples from 15 families with patients carrying a homozygous deletion of exon 7 of *SMN1*. These patients were referred for diagnosis of 5q SMA to the Research Center for Medical Genetics. Each family included one parent who was a carrier of a heterozygous deletion of exon 7 of *SMN1*, while the other parent had two copies of exon 7 of *SMN1*.

**Figure 3:**
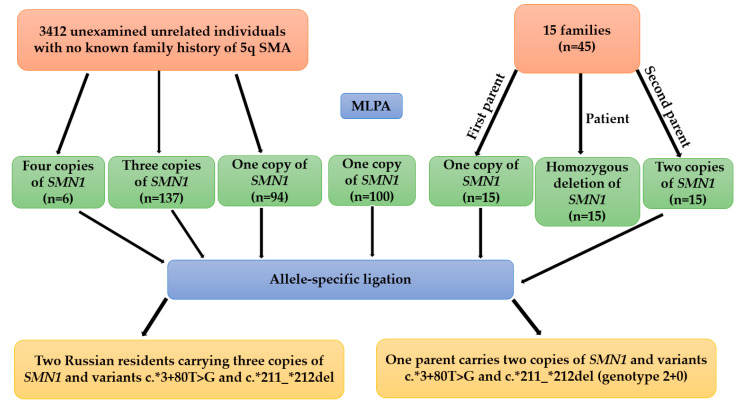
Molecular screening design of a cohort of 3412 unrelated Russian individuals and 15 families and 100 unrelated carriers of exon 7 deletion of *SMN1*.

Additional DNA samples with one copy of exon 7 of *SMN1* from 100 parents of probands with 5q SMA from the bank of the DNA diagnostic laboratory were used as a control cohort with no *SMN1* duplication (Figure 3).

Informed consent for molecular testing was obtained from all probands or their legal representatives. This study received ethical approval from the ethical committee of the Research Center for Medical Genetics, Moscow (approval number 11/1; date of approval: 23 November 2021). This study was conducted in accordance with the Declaration of Helsinki.

### 4.2. DNA Extraction

DNA was extracted from peripheral blood leukocytes using the Wizard^®^ Genomic DNA Purification Kit (Promega, Madison, WI, USA), adhering to the manufacturer’s protocol. The quality of the isolated DNA was assessed using agarose gel electrophoresis and spectrophotometric detection. The biological parental relatedness of parents with two copies of *SMN1* of patients with homozygous deletion of *SMN1* was validated using the AmpFlSTR Identifiler Direct PCR Amplification Kit (Applied Biosystems, LLC, Waltham, MA, USA) according to the manufacturer’s instructions.

### 4.3. MLPA

The copy number of exons 7 and 8 of *SMN1* and *SMN2* was detected by multiplex ligation-dependent probe amplification (MLPA): a probe system (DIALAT Ltd., Moscow, Russia) designed in the DNA diagnostic laboratory of the Research Center for Medical Genetics from 2011 to 2021 [11]. In 2021, the copy number of exons 7 and 8 of *SMN1* and *SMN2* was analyzed using the SALSA MLPA Probemix P060 SMA Carrier Kit (MRC Holland, Amsterdam, The Netherlands), according to the manufacturer’s instructions. Reaction products were analyzed via fragment analysis on an ABI Prism 3500 (Applied Biosystems, Foster City, CA, USA). For accurate quantitative analysis, the MLPA data were processed using the Coffalyser v.8 software provided by the manufacturer of MLPA (MRC Holland, Amsterdam, The Netherlands).

For detection of genetic markers of *SMN1* duplication, (c.*3+80T>G and c.*211_*212del) (Table 4) a diagnostic laboratory-designed system of probes for MLPA were added to the DNA diagnostic laboratory practice (DIALAT Ltd., Moscow, Russia). The lengths of the amplified fragments range from 104 to 114 base pairs (b.p.) (Figure 3).

**Figure 4:**
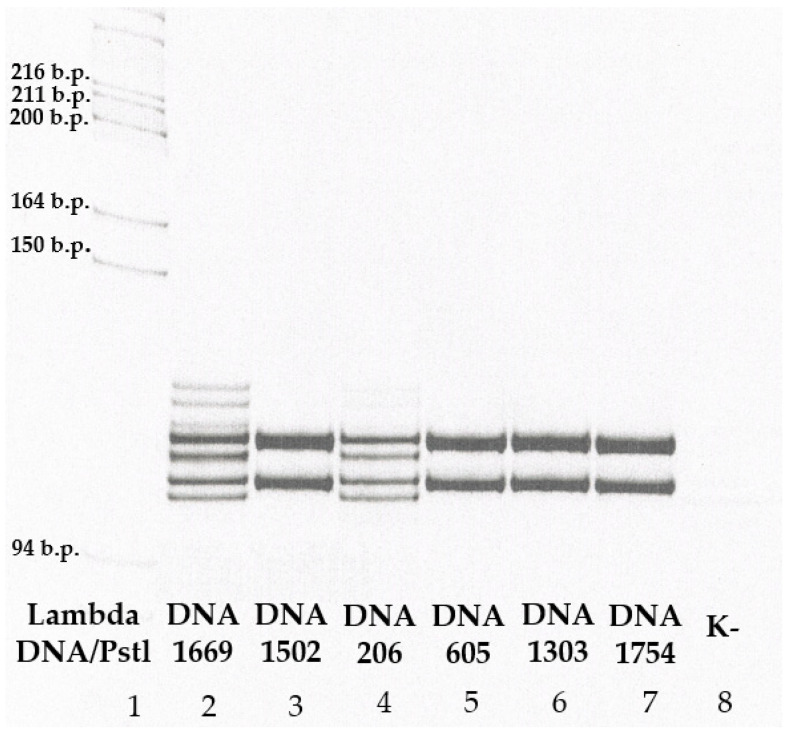
Analysis of genetic markers of *SMN1* duplication (c.*3+80T>G and c.*211_*212del). The figure shows fragments of electropherograms analyzing DNA samples (#DNA 1669, 1502, 206, 605, 1303, 1754) of Russian residents with three copies of *SMN1*. The presence of two genetic markers of *SMN1* duplication was detected in two Russian residents (track 2 and track 4), in other samples these markers were not detected. In the last track (8), there was a negative control for the reaction. The lengths of the amplified fragments range from 104 to 114 base pairs (b.p.) (Table 4).

**Figure A1:**
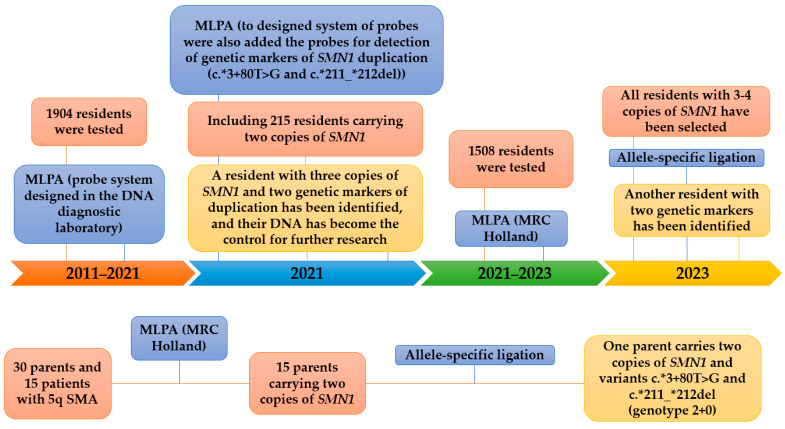
A comprehensive experimental workflow diagram.

All DNA samples of the population cohort (*n* = 3412) in which the number of *SMN1* copies were higher than 1 (*n* = 237), 30 parents of probands with 5q SMA carrying one and two copies of exon 7 of *SMN1*, and also 100 carriers of heterozygous deletion of exon 7 of *SMN1* of the control cohort were analyzed by performing of allele-specific ligation followed by visualization of the reaction results on a polyacrylamide gel using the probes described above to detect genetic markers of *SMN1* duplication (Figure 4). A comprehensive experimental workflow diagram is provided in Appendix A (Figure A1).

8 µL of ligation reaction mix contains genomic DNA and probe mix solution: MSMNC*3+80 FT, MSMNC*3+80 FG, MSMNC*3+80 R (labeled the 5′ end of the ssDNA), MRS200800214 FN, MRS200800214 FDEL, MRS200800214 R (labeled the 5′ end of the ssDNA), 1 u.a. Pfu ligase (Stratagene, La Jolla, CA, USA), and buffer (20 mM Tris-HCl (pH 7.5), 20 mM KCl, 10 mM MgCl_2_, 0.1% Igepal, 0.01 mM rATP, 1 mM DTT). The program for ligation is as follows: cycle 1 (5 min)—98 °C, cycle 2 (60 min)—60 °C. 20 µL of PCR reaction mix contains the whole volume of ligation reaction product, 2 mM dNTP, mix of universal primers 1 pm of each primer (Table 5) (DIALAT Ltd., Moscow, Russia), 1 u.a. Taq polymerase (DNA-technology, Moscow, Russia). The program for amplification is as follows: (1) initial denaturation (240 s)—95 °C, (2) denaturation (3 s)—94 °C, (3) primer annealing (3 s)—66 °C, (4) elongation (3 s)—72 °C, 34 cycles (2–4), (5) final elongation (420 s)—72 °C, (6) storage—12 °C.

### 4.4. Statistical Methods

Standard methods of statistical analysis using Statistica 10.0 software package were used to process the results obtained during the study. Data comparing multiple groups with a single characteristic were statistically analyzed using contingency table analysis with the Chi-squared test. Since the chi-square values and *p*-values are equal and indicate the presence or absence of statistical significance, both values are not consistently reported throughout the text of the article.

## 5. Conclusions

The ACMG recommends pan-ethnic screening for 5q SMA in all couples due to the high carrier frequency and the severity of the disease. Among various ethnic groups, the carrier frequency of *SMN1* deletion ranges from 1 in 25 to 1 in 50. In this study, the carrier frequency of *SMN1* exon 7 deletion in heterozygous Russian residents is 1 in 36 (95% CI 33 to 39). Additionally, the frequency of *SMN1* exon 7 duplication in Russian residents is estimated to be 1 in 25 (95% CI 20 to 30). The probability of an individual with two copies of *SMN1* carrying genotype 2 + 0 is extremely low, at 0.1%. Thus, analyzing the copy number of *SMN1* enables reliable determination of carrier status for exon 7 deletion in Russian residents with a confidence level of 99.9%. Furthermore, it has been shown that the test of the genetic markers of *SMN1* duplication, c.*3+80T>G and c.*211_*212del, provides limited informative value in Russia.

## Figures and Tables

**Table 1:** *SMN1* genotypes in the Russian population.

Genotypes	One *SMN1* Copy	Two *SMN1* Copies	Three *SMN1* Copies	Four *SMN1* Copies
*n* = 3412 (100%)	94 (2.8%) *p* < 0.001 (95% CI 91 to 97)	3175 (93%) (95% CI 3069 to 3281)	137 (4%) *p* < 0.001 (95% CI 133 to 141)	6 (0.2%) *p* < 0.001(95% CI 5 to 7)

**Table 2:** Frequency of rare genotypes of the SMN locus in population cohorts of various countries.

Ethnic Cohorts	Number of Individuals with Three or More Copies of *SMN1* in the Genotype	Number of Individuals with One Copy of *SMN1*	Study
African Americans living in the Greater New York Metropolitan area	135 of 276 (49%)	5 of 276 (2%)	[4]
Ashkenazi Jews living in the Greater New York Metropolitan area	99 of 692 (14%)	15 of 692 (2%)	[4]
Asians living in the Greater New York Metropolitan area	24 of 248 (9.8%)	2 of 250 (0.8%)	[4]
Hispanics living in the Greater New York Metropolitan area	42 of 262 (16%)	1 of 262 (0.4%)	[4]
Caucasians living in the Greater New York Metropolitan area	31 of 458 (6.8%)	12 of 458 (2.6%)	[4]
Residents of Russia	143 of 3412 (4.2%)	94 of 3412 (2.8%)	The present study
Residents of Singapore	0 of 375	6 of 375 (1.6%)	[9]

**Table 3:** Screening for the presence/absence of variants c.*3+80T>G and c.*211_*212del. Ind. = individual/s; (homo) = homozygous state; (hetero) = heterozygous state.

Variants	Parents of Patients with 5q SMA (*n* = 158)			Normal Individuals (Population Cohort)			
	One *SMN1* Copy	Two *SMN1* Copies	Three *SMN1* Copies	One *SMN1* Copy	Two *SMN1* Copies	Three *SMN1* Copies	Four *SMN1* Copies
**Cohorts of Russia (the present study)**							
Total Ind. studied	30			3412			
Ind.	15	15	0	94 *	3175 **	137	6
c.*3+80T>G	0	1	0	0	No data	2	0
c.*211_*212del	0	1	0	0	No data	2	0
**Cohorts of Spain [10]**							
Total Ind. studied	74			160			
Ind.	41	32	1	No data	99	58	3
c.*3+80T>G	1	7	0	No data	0	11	0
c.*211_*212del	1	6	0	No data	0	11	0
**Cohorts of Singapore [9]**							
Total Ind. studied	54			321			
Ind.	45	9	No data	6	315	No data	No data
c.*3+80T>G	0	3	No data	0	0	No data	No data
c.*211_*212del	0	1	No data	0	0	No data	No data
**Ashkenazi Jews living in the Greater New York Metropolitan area [4]**							
Total Ind. studied	No data			692			
Ind.	No data	No data	No data	15	315 (578) ***	99	0
c.*3+80T>G	No data	No data	No data	0	0	No data	0
c.*211_*212del	No data	No data	No data	0	0	No data	0
**African Americans living in the Greater New York Metropolitan area [4]**							
Total Ind. studied	No data			276			
Ind.	No data	No data	No data	5	136	111	24
c.*3+80T>G	No data	No data	No data	1	2 (homo) 26 (hetero)	4 (homo) 86 (hetero)	23 (hetero)
c.*211_*212del	No data	No data	No data	1	2 (homo) 26 (hetero)	4 (homo) 86 (hetero)	23 (hetero)
**Asians living in the Greater New York Metropolitan area [4]**							
Total Ind. studied	No data			248			
Ind.	No data	No data	No data	2	222	22	2
c.*3+80T>G	No data	No data	No data	0	0	2	1
c.*211_*212del	No data	No data	No data	0	0	2	1
**Hispanics living in the Greater New York Metropolitan area [4]**							
Total Ind. studied	No data			262			
Ind.	No data	No data	No data	1	219	40	2
c.*3+80T>G	No data	No data	No data	0	1 (homo) 12 (hetero)	20 (hetero)	2 (hetero)
c.*211_*212del	No data	No data	No data	0	1 (homo) 12 (hetero)	20 (hetero)	2 (hetero)
**Caucasian** **s living in the Greater New York Metropolitan area [4]**							
Total Ind. studied	No data			458			
Ind.	No data	No data	No data	12	415	27	4
c.*3+80T>G	No data	No data	No data	0	2	4	2
c.*211_*212del	No data	No data	No data	0	2	4	2

* A total of 209 individuals carrying one copy of *SMN1* were studied: 94 individuals from the population cohort, 100 individuals from the control cohort, and 15 parents of patients with 5q SMA carrying one copy. None of the genetic markers of *SMN1* duplication were detected in any of the individuals. ** In the population cohort with two copies of *SMN1*, the search for the genetic markers of *SMN1* duplication, c.*3+80T>G and c.*211_*212del, was performed in only 215 individuals. None of the genetic markers of *SMN1* duplication were detected in any of the individuals. *** Out of 578 individuals with two copies of *SMN1*, the search for genetic markers of *SMN1* duplication, c.*3+80T>G and c.*211_*212del, was performed in 315 individuals.

**Table 4:** Probes for the detection of genetic markers of *SMN1* duplication (c.*3+80T>G and c.*211_*212del). FT, FG, FN, FDEL—forward probe, R—reverse probe.

Probe Designation	Probe Sequence, 5′-3′	Length
MSMNC*3+80 (FT)	GTTCGTACGTGAATCGCGGTACGTTGGTTTGTGGAAAACAAATGTTTTTGAACAT	55
MSMNC*3+80 (FG)	GTTCGTACGTGAATCGCGGTACGGTTTGTGGAAAACAAATGTTTTTGAACAG	52
MSMNC*3+80 (R)	TTAAAAAGTTCAGATGTTAAAAAGTTGAAAGGTTAATGATGCGATCCGATGCCTTCATG	59
MRS200800214 (FN)	GTTCGTACGTGAATCGCGGTACCCAAATGCAATGTGAAATATTTTACTGGACTCTA	56
MRS200800214 (FDEL)	GTTCGTACGTGAATCGCGGTACCAAATGCAATGTGAAATATTTTACTGGACTC	53
MRS200800214 (R)	TTTTGAAAAACCATCTGTAAAAGACTGGGGATGCGATCCGATGCCTTCATG	51

**Table 5:** Sequence of universal primers for the PCR step. F—forward primer, R—reverse primer.

Primer Designation	Primer Sequence, 5′-3′
UniF (F)	GAGGAACCAGTACCCCGACATC
UniR (R)	GCCCAACATTCTATGATAGCACC

## Data Availability

The original contributions presented in this study are included in the article. Further inquiries can be directed to the corresponding author.

## Appendix B

Calculations for Hardy–Weinberg equilibrium testing are provided in detail.

p^2^ + 2 p q + q^2^ = 1 (A1)

In our study, there were 6824 chromosomes, of which 94 chromosomes had a deletion of *SMN1*.

q = 94 ÷ 6824 = 0.014 (A2)

Thus, the allele frequency of the deletion of exon 7 of *SMN1* was 0.014.

p + q = 1 → p = 1 − q (A3)

2 p q = 2 × 0.014 × (1 − 0.014) = 0.028 = 28/1000 = 1/36 (A4)

This represents a carrier frequency of approximately 1 in 36 residents.

The probability of encountering chromosomes with *SMN1* duplication in a homozygous state was calculated in our study.

137 chromosomes *SMN1* duplication/6824 chromosomes = 0.02 (A5)

2 p q = 2 × 0.02 × 0.02 = 0.0008 = 1/1250 = 5.5 (A6)

Thus, we expected to see 5–6 individuals homozygous for this duplication. In fact, 6 individuals carried 4 copies of exon 7 of *SMN1*, confirming their homozygous status for *SMN1* duplication.

q = 149 chromosomes *SMN1* duplication/6824 chromosomes = 0.022 (A7)

Thus, the duplication of exon 7 of *SMN1* was detected in 149 out of 6824 chromosomes, which resulted in an allele frequency of 0.022.

p + q = 1 → p = 1 − q (A8)

2 p q = 2 × 0.022 × (1 − 0.022) = 0.043 = 43/1000 = 1/23 (A9)

This represents a frequency of *SMN1* duplication of approximately 1 in 23 residents.

The probability of encountering the genotype 2 + 0 (in which one chromosome 5 carries deletion of *SMN1* while the other chromosome carries duplication of *SMN1*) among Russian residents was calculated in our study.

2 p q = 2 × 0.014 × 0.022 = 0.00056 = 1/1667 (A10)

Thus, the frequency of silent carriers is 0.0006, or 1 in 1667 residents of Russia.

Minor deviations from expected frequencies in population genetics studies can arise from several factors, including small population size, non-random mating, natural selection and pathogenic variants, inbreeding, genotyping errors, and the genetic heterogeneity of cohorts [12].
